# When midwife continuity of carer is the policy proposal, what is the problem of perinatal health inequalities represented to be?

**DOI:** 10.1007/s43545-025-01085-x

**Published:** 2025-05-09

**Authors:** Ditte Madsen

**Affiliations:** 1 University of Exeter (Politics), Exeter (Devon), UK; 2 Whittington Health NHS Trust, London, UK

**Keywords:** Intrapartum continuity, Carol Lee Bacchi, Social Determinants of Health, *Better Births*, Core20PLUS5, Health injustice

## Abstract

Midwife continuity of carer (MCOC) is widely recommended to protect birthing people against disrespectful care and mitigate effects of social adversity. In the UK, this is reflected in the Core20PLUS5 framework, which identifies maternity and, specifically, MCOC as one of five national priorities for reducing healthcare inequalities. Within health policy networks, the prevailing view of the policy process is that the task of government is to find solutions to existing policy problems. A critical policy approach, in contrast, considers how any policy proposal represents a problem in a particular way, shaping what is perceived as possible or desirable. Adopting this approach, I suggest that the promise of MCOC (and, specifically, intrapartum continuity) has been overstated, given the context of extreme social inequality, chronic underfunding of the NHS, the impact of austerity and the denial of structural racism in the UK. MCOC has been found to improve access, experience, and outcomes for disadvantaged and racially minoritized people by offering more personalized care. When MCOC is the policy proposal, however, the problem of inequalities in perinatal health tends to be represented in terms of fragmented maternity care, limiting policy discussion of the drivers of health inequality. This enables elected officials to appear to address health inequality by relying on midwives to adopt more flexible working practices. Representing the problem as a matter of health justice, in contrast, not only requires that personalized care is informed by structural competency and cultural safety but demands that policymakers address structural drivers beyond the healthcare system.

In the United Kingdom, health inequalities associated with the perinatal period are starkly demonstrated by maternal deaths. Maternal mortality rates are increasing overall while also widening between those living in deprived areas and those living in affluent areas (Knight et al. 2022). With suicide a leading direct cause of death and increasing threefold between 2019 and 2022, birthing people faced with social disadvantage are unequally exposed to avoidable death (Knight et al. 2022). 22% of those who died between 2020 and 2022 were known to social services (Felker et al. 2024). Meanwhile, Black and Asian birthing people remain respectively three and two times as likely to die compared with white birthing people (Felker et al. 2024). The rate of stillbirth and neonatal mortality varies too by ethnicity and level of deprivation (Knight et al. 2022). Babies of Asian and Black ethnicity have much higher mortality rates than babies of white ethnicity, with Black babies more than twice as likely to be stillborn than white babies (Knight et al. 2022). Babies born to mothers who live in areas affected by socio-economic deprivation are twice as likely to die in the neonatal period as those from the least deprived areas (Knight et al. 2022). Importantly, babies of Black and Asian ethnicity are more likely to also be impacted by the higher rates of stillbirth and neonatal death that is associated with socioeconomic deprivation (Felker et al. 2024; Jardine et al. 2021). These devastating outcomes reflect a much wider problem of inequalities in experience as revealed by several important reports, which demonstrate the extent to which minoritized and disadvantaged communities have poorer experiences of perinatal care that often causes them to disengage or leaves them traumatized (APPG Birth Trauma 2024; Birthrights and Birth Companions 2019; Birthrights 2022; Peter and Wheeler 2022).

In response to this alarming situation, targeted provision of Midwife Continuity of Carer (MCOC) has been proposed to reduce inequalities in access, experience, and outcomes in perinatal care (Hadebe et al. 2021; NHS England 2021a; Rayment-Jones et al. 2023; RCM 2024). As defined in the Cochrane Review, MCOC models ‘provide care from the same midwife or team of midwives during pregnancy, birth, and the early parenting period in collaboration with obstetric and specialist teams when required’ (Sandall et al. 2024). Significantly, levels of continuity are measured according to whether a known carer is present at birth (intrapartum continuity) (Sandall et al. 2024). MCOC is understood to protect against disrespectful care since birthing people are more likely to report feeling listened to when cared for by a person with whom they have built a relationship, with midwives taking greater responsibility and birthing people having improved trust and confidence in the midwife to disclose concerns (Rayment-Jones et al. 2023; Sandall et al. 2024). MCOC is understood to mitigate the impact of social adversity through midwives having knowledge of an individual’s medical and social history, providing more opportunities for social support from multi-disciplinary services and earlier referral (Rayment-Jones et al. 2023; Sandall et al. 2024). While clinical research on MCOC emphasizes that the model requires adequate staffing to be safely implemented, it also highlights its potential as a cost-saving policy (Rayment-Jones et al. 2023; Sandall et al. 2024).

The current policy priority of providing enhanced maternity care for the most disadvantaged and minoritized is a positive development. However, it is important to consider how this policy proposal shapes and limits what we perceive the problem of perinatal health inequalities to be (Kemm 2006). Speaking at a national conference on *Tackling Inequality* in 2023, Prof Helen Stokes-Lampard (Chair, Academy Medical Royal Colleges) highlighted the need to broaden the range of research evidence informing strategies to address health inequalities by drawing on insights from social science. Middlemiss et al. (2024) have similarly called for existing evidence that supports MCOC to be critically examined in relation to the political and structural contexts in which the impetus for its implementation has developed (cf. Ashley 2016). This review paper contributes to this by considering MCOC from a critical policy perspective, which works ‘backwards’ from policy proposals to ‘put into question their underlying premises, to show that they have a history, and to insist on questioning their implications’ (Bacchi and Goodwin 2016, p. 16; cf. Fischer et al. 2015). Reflecting a wider public understanding, clinical researchers in midwifery studies tend to see policy as about finding solutions to existing problems. In contrast, critical policy scholars highlight how policy solutions and problem definitions are inseparable in practice (Bacchi 1999; Bacchi and Goodwin 2016). This is because any policy proposal necessarily represents the problem it addresses in a particular way by providing a language with which to talk and think about it while obscuring competing representations. By asking “what’s the problem represented to be?,” critical policy analysis aims to make the politics of the policy process visible by identifying the *presuppositions* and *effects* of any policy proposal, investigating the context in which it is articulated, the discourse in terms of which a problem is framed and the material consequences that follow from it (Bacchi 1999, 7–10; Bacchi and Goodwin 2016). Moreover, it considers *what is left unproblematic* in any given problem-representation and how policy responses might differ if a *problem were represented differently* (Bacchi 1999, p. 13).

In this review paper, I argue that, when MCOC is the policy proposal, the problem of perinatal health inequalities tends to be represented in terms of the fragmentation of maternity care. By contextualizing the current focus on the targeted provision of MCOC in relation to the wider political and historical context within which the proposal for its universal provision was developed in the UK, I discuss how this problem-representation *presupposes* that inequalities in perinatal health can be addressed through reorganization of maternity care. In being promoted as cost-neutral to implement and, ultimately, cost-saving to government, it underestimates the adverse *effects* of the policy on midwives, who it relies on to take greater responsibility and work flexibly by being available at times of high activity and rest when activity is low. In attributing the problem of minoritized and disadvantaged birthing people not being heard primarily to models of care, it tends to *leave unproblemati*c how wider government policies, such as austerity and the hostile environment, contribute to what Marmot (2015) calls the ‘causes of the causes’ of ill health and reduced life expectancy (Marmot et al. 2020). When the *problem is represented differently*, as a matter of health justice, I argue, any policy response requires not only that disrespectful care is reduced through training *all* healthcare professionals in structural competency and cultural safety, but that policymakers’ attention is directed beyond the healthcare system to the structural drivers that expose people to ill health and avoidable death.

By reviewing the evidence-base that supports the targeted provision of MCOC, in the first section, I show how representing the policy problem as ‘fragmented care’ relies on contrasting midwife-led MCOC with ‘other models of care’ (including obstetrician-led and family-doctor-led care), which do not reflect the common work practice of midwife-led practice in the UK (in which continuity is provided during the antenatal and postnatal periods, while care for labour and birth is provided by a labour ward midwife). Rather than intrapartum continuity, I suggest, the key mechanism that improves outcomes and experiences for birthing people might be the enhanced time available for midwives to build relationships with women. While enhancing the amount of time midwives can spend with the most disadvantaged women at this critical time in their lives is crucial, this raises the question of how this enhanced provision of midwifery care is resourced when scaled up. By contextualizing the current policy proposal for the targeted provision of MCOC in relation to *Better Births* and the failed attempt to implement the universal provision of MCOC, in the second section, I show how the burden of realizing the promise of MCOC depends on midwives working more flexibly in response to birthing people’s urgent and unpredictable needs over long periods of time. In the concluding section, I argue that the prioritization of MCOC risks displacing the policy focus away from the structural determinants of health. Representing the problem as a matter of health injustice, in contrast, demands not only that all healthcare professionals provide rights-respecting care that is informed by structural competence and based on cultural safety but that we reframe how we talk about health to raise public and professional awareness of where meaningful intervention on health inequality lies beyond the healthcare system.

## Making time for birthing people within the midwife continuity of carer model

After many decades of robust evidence about the scale of inequality in health in the UK, reducing health inequalities is finally on the agenda within the NHS (Acheson 1998; DHSS 1980; Marmot 2015; Marmot et al. 2020). This is evident in Core20PLUS5, which sets out the NHS England approach to tackling healthcare inequalities through targeted interventions for the most disadvantaged across five national priority areas (NHS England 2021a). In maternity, Core20PLUS5 singles out midwife continuity of carer (MCOC) as the policy priority for addressing health inequalities associated with the perinatal period. According to NHS England, this ‘relationship between care giver and receiver has been proven to lead to better outcomes and safety for the woman and baby, as well as offering a more positive and personal experience’ (NHS England 2021b). However, as I show in this section, the evidence-base for the MCOC model is not as clear as presupposed within health policy networks because it relies on comparing MCOC with ‘other models of care’ (including obstetrician-provided care and family-doctor provided care) that often do not reflect working practices in the UK (Sandall et al. 2024). While the scholarly literature recognizes that the underlying mechanisms of MCOC that lead to improved outcomes and experiences are complex and not fully understood (Rayment-Jones et al. 2023), the reason to critically reflect on the benefits attributable to intrapartum continuity, specifically, is that, although this is the most demanding aspect of the policy proposal for midwives, it is taken to be the measure of whether relational continuity is achieved. In addition to critically analysing the evidence base for MCOC, I situate this research within the wider policy-making process by engaging with it not only according to the norms of evidence-based medicine but by also interpreting this research itself as a discursive activity.

The current policy prioritization of targeted provision of MCOC to address inequalities in perinatal health is reflected in national strategy (CORE20Plus5), which aims to provide MCOC for the 20% most deprived and those belonging to racially minoritized groups known to be at increased risk of experiencing poor outcomes (NHS England 2021a). It is also evident in a tool developed by the Royal College of Midwives to standardize the assessment, recording of and response to social complexity (RCM 2024), which recommends MCOC for all levels of care in response to social risk factors beyond those who are thriving and whose needs are met by universal care.^1^ While the current policy priority of targeting enhanced maternity care to the most disadvantaged is welcome, the policy agenda to implement MCOC in England is not new. Following the recommendation of the *Better Births* report in 2016, ambitious policy aims were established in 2021 to make MCOC the default model of care offered to all (National Maternity Review 2016; NHS England 2021b). Yet implementation of the universal provision of MCOC was soon paused in response to the Ockendon report, which found that the implementation of MCOC compromised safety in the absence of adequate funding and in the context of a severe staffing crisis (Independent Maternity Review 2022).

This is because MCOC, which includes intrapartum continuity, is difficult to implement due to the unpredictability of labour and birth, which makes it necessary for midwives to change how we work to be available when needed as opposed to working according to a roster, which is planned. Where MCOC has been implemented, heads of midwifery (HoMs) have reported significant impact on the remaining clinical areas. 73% of HoMs reported MCOC was difficult or very difficult to implement while a further 11% of HoMs had not attempted to implement MCOC (RCM 2019). Among the barriers cited were lack of additional resource to backfill areas left by those joining the MCOC models, and the high importance placed on midwifery work/life balance, childcare, set shifts and historic working patterns, and staff working part-time (McCourt et al. 2023; RCM, 2019). Despite the known challenges of implementing MCOC, its upscaling is high on the maternity agenda, with the NHSE National Lead for Continuity calling for MCOC to be implemented at speed and scale as a moral imperative (Maternity & Midwifery Forum 2023). The prioritization of MCOC as a policy response to health inequalities is also reflected in scholarly research with Styles et al. (2020, 343), for instance, asserting that MCOC ‘is arguably the most significant factor in enhancing women’s clinical outcomes during childbearing and facilitating a positive childbirth experience’ (cf. Dahlen et al. 2022; e951; McCourt et al. 2023, p. 6; McInnes et al. 2020; Rayment-Jones et al. 2023).

Given the difficulties of implementing MCOC, and, especially, the challenges posed by intrapartum continuity, however, it is essential that we evaluate the evidence cited in relation to the impact of this aspect of care as well as the effects of MCOC as a policy discourse (Middlemiss et al. 2024). Reflecting on the policy commitment that 75% of Black women should be provided with MCOC by 2024, for instance, leading Black maternal health advocate, Sandra Igwe (2022, 26) reflects that it would have been a ‘nightmare come true’ if she had been promised continuity of care from the ‘same midwife who was cold and treated [her] differently because of [her] race.’^2^ While clinical research on MCOC has shown its potential to improve rights-respecting care (Rayment-Jones et al. 2023; Sandall et al. 2024), Igwe’s reflection on her own experience calls attention to how the policy proposal of MCOC itself shapes what the problem of perinatal health inequalities is represented to be (Bacchi and Goodwin 2016, p. 17). MCOC promises to ensure that individuals are heard and understood because care is informed by a broader understanding of a person’s life and the choices and support available to them within their social context. Within this problematization, however, as Igwe’s observation highlights, the birthing person is reliant on the protection and voice of a known midwife to be heard and respected by all healthcare professionals at the time of birth. In doing so, this problem-representation omits how structural racism is making people sick long before they enter the healthcare system and how the wider policy context is producing interpersonal and institutional racism within and beyond the healthcare system (e.g. through maternity charging and the mainstreaming of far-right anti-immigration discourse).

Within policy networks, researchers not only contribute to the solution of existing problems but also to the representation of policy problems (Bacchi and Goodwin 2016). This is apparent in how clinical researchers typically represent the problem to which the policy proposal of MCOC is a response as *fragmented care* (Hainsworth et al. 2021; Perriman and Davis 2016; Psaila et al. 2014). For instance, Rayment-Jones et al. (2016 – emphasis added) observe that MCOC has ‘long been associated with increased trust between a woman and midwife, whereas *fragmented*, *industrialized models of maternity care* are far from conducive for the development of trust,’ McInnes et al. (2020, 2 – emphasis added) state that, despite our knowledge that MCOC is a ‘superior model of care, the known risks associated with *fragmented care* and ongoing efforts to drive change, its uptake is slow and patchy’ and Rayment-Jones et al. (2020; e315 – emphasis added) hypothesize that ‘the current *fragmented service* women experience when accessing maternity care is directly linked to…health inequalities.’^3^ The purpose of examining how academic researchers contribute to policymaking as a discursive activity in this way is to make visible how the representation of the problem of perinatal health inequalities as ‘fragmented care’ contributes to everyday understanding within wider policy networks, limiting what can be talked about by structuring arguments and constituting objects and subjects in language (Bacchi 1999, 39–49). Importantly, as I discuss below, it is these objects and subjects that become the targets for policy intervention and governing (Bacchi and Goodwin 2016).

Significantly, the characterization of MCOC in contrast to fragmented care relies on previous research, which compared the effectiveness of MCOC to other models of care (see Davey et al. 2005; Hildingsson et al. 2002; RCM 2014; Van Walraven et al. 2010). In particular, the 2016 Cochrane review (Sandall et al. 2016b), which involved 17,674 women, was seen to have definitively settled questions relating to the benefits of MCOC (Middlemiss et al. 2024). However, the distinction between MCOC and ‘other models of care’ which the Cochrane review relied on, should be treated with caution. While the review has since been updated (Sandall et al. 2024), this distinction between MCOC and other models of care remains unchanged. The original review included fifteen research trials comparing outcomes for individuals who received MCOC with those receiving ‘other models of care.’ It found that MCOC impacted positively on clinical outcomes, including reduced risk of preterm birth, pregnancy loss and interventions during birth. However, the revised review (which included seventeen studies involving 18,532 women) concluded that MCOC had little or no effect on fetal loss or pre-term birth (Sandall et al. 2024). In both reviews, birthing people who received MCOC reported greater levels of satisfaction with various aspects of care (Sandall et al. 2016b, 2024). The other models to which MCOC is contrasted, in both reviews, are obstetrician-provided care, family-doctor provided care, and shared models of care. Yet, as the authors of the original report recognize, both obstetrician-led and family-doctor-led care, in which midwives work in supportive, non-decision-making roles, are rare in the UK (Sandall et al. 2016b: 7; Taylor et al. 2019). In the updated review (Sandall et al. 2024), only 7 of the 17 randomized controlled trials reviewed were conducted in the UK with just one of these conducted in the last twenty years (Fernandez Turienzo et al. 2021a).^4^ Consequently, neither the original nor the updated review investigates the difference between MCOC and existing models of midwife-led maternity care in the UK in which continuity is provided during the antenatal and postnatal periods, while care for labour and birth is provided by a labour ward midwife. The main benefit of this existing model is that it significantly reduces the requirement for midwives working on-call, with the associated benefit of increased acceptability to midwives.

The current focus on providing MCOC to the most disadvantaged and minoritized birthing people rightly seeks to address the discrimination and mitigate the social adversity faced by these groups during pregnancy and early parenthood, who are less likely to be listened to and less likely to be referred to and taken on by services such as specialist perinatal mental health teams (Redshaw and Henderson 2016). This reproduces ‘the inverse care law’ first described by Hart (1971) in that living with deprivation or as a minoritized person is associated with increased risk of mental health difficulties while also limiting access to services. In this context, MCOC has been found to improve outcomes for the most disadvantaged. An observational cohort study of women receiving caseload midwifery care in inner city London, for instance, found that this significantly reduced preterm birth and birth by caesarean section when compared with traditional care (whether midwifery-led or consultant led/shared care) (Hadebe et al. 2021).

The study by Hadebe et al. (2021) demonstrates the significant impact that targeted and well-resourced midwife-led care, such as that provided by the Lambeth Early Action Partnership (LEAP), can have. However, as the authors observe, the key to this is midwives having ‘time with women to build trust and rapport, to observe a woman’s surroundings and assess what risks have not been verbalized, to establish solutions that tailor into a woman’s framework and community’ (Hadebe et al. 2021, p. 10). This includes allowing time for conversations about birth but also about wider aspects of the person’s life. Time appears as a crucial variable that is likely to significantly impact on improving outcomes by enabling regular, longer appointments, home visits, conversations about services and making referrals. Some of the twelve MCOC midwives participating in focus groups conducted by Rayment-Jones et al. (2020, 6), for instance, identified the importance of time to build trust and advocate for birthing people with one midwife reporting that ‘having the time to do that, definitely as a traditional midwife you wouldn’t have the time to do that.’ Another midwife said: ‘it was all very routine, and everything was normal, and I was thinking, oh like it’s a really quick appointment compared to normal, so I said to her, ‘How’s everything? Like how’s your housing going, um, how’s everything at home?’ and then she opened up…that I wouldn’t have discussed if…I’d had a 20-minute appointment’ (Rayment-Jones et al. 2020, 6). The community-based setting in which a service is provided has also been identified as a potentially important factor in improving outcomes (Hadebe et al. 2021, p. 11; Fernandez Turienzo et al. 2021). In other words, while there is some evidence that MCOC improves outcomes for the most disadvantaged birthing people, this might be more attributable to the enhanced level of midwifery care that is provided within the well-resourced small-scale pilots on which the evidence is based rather than ensuring intrapartum continuity.

Evidence that the provision of MCOC improves experiences of health care for the most disadvantaged might similarly be attributable to the enhanced levels of service in pilots of MCOC. For instance, the qualitative study cited above, which evaluated two specialist models of care in London targeting women with low socioeconomic status and social risk factors, identified a range of specific maternity care mechanisms whereby MCOC might reduce health inequalities (Rayment-Jones et al. 2023). The authors of the study rightly highlight the importance of understanding how inequality is experienced through everyday interactions that produce social suffering through discriminatory, impersonal, and stigmatizing maternity care (Rayment-Jones et al. 2023; Frost and Hoggett 2008). The experiences recounted, in fact, represent violations of human rights, such as being dismissed as not in labour with subsequent unattended birth, not receiving pain relief when requested, not being given information or the opportunity to discuss and plan care, and caregivers relying on racist stereotypes (cf. APPG Birth Trauma 2024; Birthrights and Birth Companions 2019; Birthrights 2022; Peter and Wheeler 2022).

MCOC is seen to protect against disrespectful, paternalistic and sometimes abusive care otherwise encountered within a fragmented health care system since it is associated with being listened to, a sense of safety to disclose and escalate concerns, reduced fear of social care involvement and in turn reduced experience of being monitored, as new parents reported feeling able and more confident to demonstrate parenting ability (Clayton et al. 2022, p. 3; Rayment-Jones et al. 2023; e320-321). Increased trust and confidence through getting to know a named midwife was valued and sought, reducing risk of disengagement, for instance, where maternity services are perceived as a threat that could lead to the removal of their child, including a greater sense of being supported emotionally with care experienced as more holistic and less medicalized. MCOC was also seen to mitigate the impact of social adversity through midwives having knowledge of an individual’s medical and social history, which leads to a greater understanding of complexity of needs and more personalized care. Information giving is reported to be more tailored and meaningful, based on individual needs and stage of pregnancy, which is seen as contributing to better preparation for birth, reduced anxiety, sense of control and confidence in ability to parent, increased breast-feeding initiation, and potentially improved child protection outcomes. Improved antenatal education and information by a known midwife was seen to contribute to safer maternity care by positively affecting sense of access and encouraging timely help-seeking when concerns arose while also connecting families with local support, such as that relating to parenting, and breastfeeding (Rayment-Jones et al. 2023; e323).

Continuing to contrast MCOC to fragmented care, however, risks reproducing the limitations of the Cochrane Review, which did not investigate the benefits of midwife-led models that did not include intrapartum continuity since these were grouped together with obstetrician-provided care and family-doctor provided care as ‘other models.’ Indeed, the scholarly literature that recommends MCOC to reduce inequalities in perinatal health often conflates the significance of intrapartum continuity with the crucial role that access to skilled and competent midwives plays in improving maternal and neonatal health across the world. In doing so, it overstates the promise of MCOC in reducing adverse outcomes. In a comment in *The Lancet*, for example, Dahlen et al. (2022; e951) claim that ‘relational models of care such as continuity of midwifery care…have the potential to save 4.3 million lives per year.’ However, the study that they cite (Nove et al. 2021) predicted not that MCOC, specifically, but rather ‘midwife delivered interventions,’ more generally, are key to improving maternal and neonatal health globally. The authors go on to suggest that not offering continuity of carer reflects the low status of midwives and, more broadly, the low value of women in society. While they rightly recognize how the low status of the role of the midwife reflects a broader lack of value and investment in women’s health and education, they mistakenly move from evidence about the benefits of midwife delivered interventions to their claim about the urgent need to upscale MCOC.

On the contrary, as I will argue in the next section, the policy focus on MCOC reflects the devaluation of female professions. This is because a significant effect of the policy proposal of MCOC in response to the problem of ‘fragmented care’ is that it relies on midwives to adopt flexible working practices required to provide intrapartum continuity. By constructing the policy problem as fragmented care, policy discourse around MCOC produces the ideal subject of the midwife as caring and flexible while characterizing those unable or unwilling to adopt the MCOC model as inflexible and resistant to change. Indeed, the frequent observation that midwives are often willing to go ‘above and beyond’ when working within the MCOC model due to the job satisfaction it provides arguably relies on the exploitation of emotional labour. Contextualizing the evidence base supporting the targeted provision of MCOC to address perinatal health inequalities in relation to the historical and political context of *Better Births* in which the policy proposal for the universal provision of MCOC was previously adopted, thus foregrounds that a significant effect of representing the policy problem in terms of fragmented care is to place the burden of mitigating health inequalities on midwives.

## Remembering *Better Births* and the impact on midwives of implementing MCOC

In line with the prioritization of MCOC within health policy networks, clinical researchers are recommending that the targeted provision of MCOC be implemented, upscaled and sustained to tackle inequalities (Hadebe et al. 2021, p. 2; Dahlen et al. 2022; e951; Rayment-Jones et al. 2023; McCourt et al. 2023). Clinical researchers acknowledge that MCOC is ‘not a panacea for all poor health and social outcomes’ and that the outcomes of pilot studies demonstrate what could be achieved in a well-resourced workforce (Rayment-Jones et al. 2020, p. 9). However, in viewing their role as providing evidence-based recommendations to address existing policy problems, they tend not to recognize how they participate in the policy-making process by shaping how policy problems are represented (Bacchi 1999; Kemm 2006, p. 322). As a policy proposal, MCOC is attractive to governments since it promises an enhanced service through reorganization, making it compatible with the politics of austerity. However, as the Ockendon inquiry starkly demonstrated, and heads of midwifery know (RCM, 2019), the reality for midwives is that the implementation of MCOC adds more pressure to an already over-stretched service. It is therefore important to situate the current policy recommendation for the targeted provision of MCOC in the political context of *Better Births*, which proposed the universal provision of MCOC in response to safety concerns, which were similarly represented in terms of fragmented care. As I discuss in this section, *Better Births* was a policy high in symbolic value, but which provided no further investment in maternity services since the authors of the report argued that increasing workforce numbers would not benefit the aim of continuity (National Maternity Review 2016, 96; cf. Schneider and Ingram 1993). Instead, the success of the policy relied on increasing midwives’ sense of responsibility, commitment, and personal fulfilment through work as dedicated and passionate caregivers.

The drive for woman-centred, personalized maternity care is not new but was the overriding objective of changes proposed in the 1993 Cumberlege report, *Changing Childbirth*, which called for continuity of care, choice, and control for women (Department of Health 1993; see McIntosh et al. 2014). Following the Morecambe Bay investigation into the high number of deaths of mothers and babies in one NHS maternity unit in 2015, MCOC was again promoted in response to the problem of safety for all birthing people. The Kirkup report identified systemic and lethal failures within the maternity unit, including, where midwives were concerned, ‘poor clinical competence, insufficient recognition of risk, inappropriate pursuit of normal childbirth and failures of team-working’ (Kirkup 2015, p. 8). Alarmingly, this included a failure to listen to women when they report concerns about their own or their baby’s health.^5^ This became a driver for the transformation of maternity services, with the problem of birthing people not being listened to represented in terms of fragmented care and the solution found in improving the relationships between midwives and birthing people through MCOC. In 2016, the Sheila Kitzinger symposium published an influential report, which proposed scaling up and implementing MCOC as a cost-neutral policy. This was to be achieved by increasing the flexibility of midwives’ working pattern and thereby facilitating the matching of the time midwives work to women’s needs (Sandall et al. 2016a, 7–10). Given the unpredictability of the timing and length of labour and birth, the report stressed the need for midwives to ‘work extra hard during the peaks, and rest during the lulls’ (Sandall et al. 2016a, p. 23, 7).^6^

The *Better Births* report had the laudable aim of making maternity services in the UK safer and more responsive to women’s choices (National Maternity Review 2016). *Better Births* received broad support from midwives and advocacy organizations since it provided a vision of midwife-led maternity care, centred on the needs of birthing people, which recognized the importance of personal relationships. For instance, Chief Executive of the Royal College of Midwives, Warwick (2016) welcomed the report as a ‘triumph for women.’ Alongside MCOC, the review recommended the introduction of personalized maternity care budgets to ensure that women are in control of their own care. Each birthing person would have a budget, which could be spent on the services they decided are most appropriate to their situation. While this aspect of the policy proposal was not implemented, it indicates the wider political context within which the proposal for the universal provision of MCOC was articulated. In particular, the association of personalized care with consumer choice was consistent with the independent chair of the review’s long-standing advocacy of private provision and charging within the NHS (see Ashley 2016; Lister 2016; Middlemiss et al. 2024, p. 9).^7^ MCOC subsequently became the defining policy proposal of *Better Birth*s, according to which, ‘women should have continuity of the person looking after them during their maternity journey, before, during and after the birth’ in contrast to the existing model that was represented as fragmented and delivered within ‘hierarchical, bureaucratic organizational structures’ (National Maternity Review 2016, 3, 77). *Better Births* therefore aimed to enhance the responsiveness of maternity services without any increase in the midwifery workforce (National Maternity Review 2016, 96). It promised a safer and more responsive maternity service by restructuring the way midwives work.

As such, *Better Births* can be seen as a symbolic policy, which enabled elected political officials to benefit from aligning their interest with those of mothers and families and health care professionals, while offering little in the way of resources (Schneider and Ingram 1993, p. 334). Rather than investing in maternity services, *Better Births* was based on reorganization, which promised an increasingly flexible caring workforce.^8^ In this way, the policy proposal shifted the burden of delivering safer and more personalized care to midwives in a context of chronic staff shortages with many in the profession leaving or intending to leave (Hunter et al. 2017). The burden imposed on midwives to implement *Better Births* was obscured by the gendered image of the midwife as caregiver. The policy aimed to make maternity services more responsive by promoting ‘close professional friendship’ between midwives and women and celebrated the ‘passion and dedication’ of midwives (National Maternity Review 2016, 2). In this way, the policy celebrates midwives as caring, other-regarding and responsive while suggesting that midwives should be willing and readily available to meet the needs of birthing people. A similar emphasis on midwife flexibility and generosity is evident in much of the research on MCOC, with Crowther (2017, 1), for example, observing that ‘the emphasis on flexibility, generosity, working together, self-determination and a focus on self-care is crucial’ for MCOC.^9^*Better Births* promoted MCOC as a means of increasing midwives’ autonomy over their work and presented the sense of personal obligation felt by midwives.^10^*Better Births* suggested that mechanisms of ‘advocacy, trust, choice, control and listening to women’ are responsible for enhanced job satisfaction, with midwives working within MCOC models ‘oriented towards the woman and her community, rather than towards the needs of the institution’ (National Maternity Review 2016, 10). Finlay and Sandall (2009, 1229) have similarly proposed that MCOC works as a means of ‘enhancing the health professional’s ‘exercise of discretion’ within bureaucratic healthcare systems.^11^ However, this is contradicted by evidence that most midwives are unable or unwilling to work under MCOC (Taylor et al. 2019, p. 4).^12^

While *Better Births* presented MCOC to midwives as an opportunity for self-discovery and development, the gendered language in which it is framed can, in fact, be seen to encourage midwives’ self-effacement while discouraging dissent. *Better Births’* vision relied on the increased availability of midwives to provide intrapartum continuity, shifting from a language of midwives being on-call with adequate remuneration to simply being available. This vision is often reflected in current scholarly research on MCOC and health inequality, which relies on midwives developing passion and commitment, so they are driven to work excess hours to be available for people on their caseload. Several studies have found that the increased sense of responsibility experienced by midwives working within MCOC models is a motivating force leading to increased commitment and driving a desire to go above and beyond (Clayton et al. 2022; Leavy and Leggett 2022). In their recent review of evidence relating to midwives’ experience of working in MCOC models, for instance, Leavy and Leggett (2022) found that reports of midwives working on days off and taking extra on-calls were common. Midwives are encouraged to believe that a positive outcome for the birthing person depends on them being available to be present at the birth. As one midwife observed: ‘I probably showed them a model that was too balanced towards just giving everything… (I wish I had) real time off and done more things with the family and showed them that there was life where the phone wasn’t always there and the possibility of mum always having to rush away’ (Midwife cited in Leavy and Leggett 2022, p. 6). While being available for disadvantaged and minoritized birthing people is admirable, the requirement for intrapartum continuity blurs the division of work and leisure time and encourages a culture of self-sacrifice so that midwives are likely to feel guilt when unavailable (Stoll and Gallagher 2019).

The current policy focus on targeted MCOC for the most disadvantaged and minoritized birthing people therefore reproduces many of the assumptions about midwives that underpinned *Better Births*. Resistance and concern among midwives are often reported as barriers to be overcome through effective leadership and communication (McCourt et al. 2023, p. 4). For instance, Taylor et al. (2019, 10) warn that the negative effects of midwife resistance to MCOC in terms of relationships and safety ‘should not be underestimated.’ They hypothesize that midwives who do not volunteer for MCOC working may have less ‘philosophical commitment to physiological pregnancy and birth or woman-centred care or have different personal attributes’ (Taylor et al. 2019, p. 10). In addition, they propose that providing all aspects of care through pregnancy, birth and postnatal, ‘has the potential to change midwives through personal and professional development, accountability, responsibility, reflective practice and being in control of their own diary and workload’ (Taylor et al. 2019, p. 10). Meanwhile, they suggest that resistance to offering MCOC is reflective of midwives’ beliefs, biases, and fears. This is problematic, to say the least, and ignores midwives’ knowledge and expertise, including previous experience of working in integrated care models and effects of working flexibly and on-call on their own mental and physical health, their quality of life and its effect on their families.

While the current midwifery staffing crisis is recognized within the literature as one of the main barriers to implementing MCOC, the model is more often portrayed as part of the solution to the staffing crisis rather than recognized as an exacerbating factor (McInnes et al. 2020).^13^ In contrast to larger studies where evidence suggests that midwives would consider leaving if compelled to adopt MCOC (Taylor et al. 2019; RCM 2019), the evidence supporting the viability of MCOC is based on relatively new pilots with sustainability over time not established (Thorpe 2022).^14^ There is evidence that job satisfaction of midwives who have chosen to work in MCOC models has increased while indicators of stress and burnout have reduced (Cramer and Hunter 2019; Dawson et al. 2018; Jepsen et al. 2017; Styles et al. 2020). Reasons identified within the literature for increased levels of job satisfaction include enabling midwives: to get to know and develop meaningful relationships with service users; to use the full range of their skills throughout all stages of pregnancy, birth and postnatal; to become more passionate about ensuring high quality care as they build relationships; to improve working relationships among colleagues through shared commitment to making the model work, and; to do their job more easily due to knowing a person’s history well and being aware of what support is available locally. However, due to its reliance on a self-selecting group of midwives, who have participated in well-funded and successful pilot MCOC projects, the research tends to ignore the structural factors that shape job satisfaction and well-being among midwives. It is well known that, within health care, high workload, and understaffing correlate with burnout (Cramer and Hunter 2019; Kinman et al. 2020). The increased satisfaction of midwives participating in pilot studies may therefore be attributable to having more time and smaller caseloads, which contributes to the benefits emphasized in the research. Significantly, Dharni et al. (2021) found similarly high levels of satisfaction among both users and midwives within midwifery-led continuity of carer that did not include intrapartum continuity. The mechanisms identified by the researchers were factors related to structure and resource, namely the combination of additional time and smaller caseloads within the model offering continuity during pregnancy and following birth. This is important, as intrapartum continuity requires midwives being available and on-call with unpredictable working patterns that are not acceptable to many midwives and are known to be associated with burnout and stress and an obstacle to the sustainability of the continuity model (Green et al. 2000: 195).

While the policy prioritisation of MCOC to address perinatal health inequalities is based on the success of well-resourced and small-scale pilot projects staffed by self-selected midwives who can work flexibly, researchers have paid little attention to attempts to establish local services based on MCOC that have not been sustained. The Jubilee team in Plymouth, for instance, was held up as an example of how MCOC might be successfully implemented at a meeting of national leaders in 2018 to support local maternity providers in delivering the *Better Births* agenda.^15^ At the meeting, the issue of retention was raised by a local midwife but not addressed, even though it was already apparent that there were difficulties sustaining this service and it was subsequently withdrawn.^16^ Neighbourhood Midwives in London, established as an employee-owned two-year pilot scheme to deliver MCOC to women with low-risk pregnancy in Waltham Forest in London, was also promoted as an exemplar of MCOC. Neighbourhood Midwives was launched with private investment as an independent midwifery provider in 2013 with the intention to be commissioned by the NHS (Moorhead 2012). Speaking at an event in 2017 to mark the first year of service, Baroness Julia Cumberlege applauded the success of the service saying that others should learn from it (O’Callaghan 2017). However, by 2019 this service had been abruptly disbanded without public explanation, leaving those birthing people affected by the closure feeling let down and the local MP calling its sudden closure a mystery (Sherwood 2019). Speaking in 2020, former CEO Annie Francis (who was a member of the national maternity review team led by Julia Cumberlege) discussed how the Maternity Choice and Personalization Pioneers program introduced through *Better Births* had provided an opportunity for the service to be established as a non-NHS provider (Walker 2017). In contrast to what Francis (2020) described as a medicalized model and an ‘old-fashioned style of management’ based on ‘command and control’ within the NHS, Neighbourhood Midwives sought to create ‘safe circles’ for midwives outside the NHS, based on self-management with everyone working together and bringing their ‘whole self to work.’ Despite the cultivation of alternative ways of working, however, tensions emerged requiring resolution with the help of external consultants. A notable source of tension concerned the sustainability of flexible work practices, which required midwives to be on-call 24/7 for 40 weeks of the year to ensure intrapartum continuity. While Francis (2020) pointed to the success of Neighbourhood Midwives in terms of outcomes, the model had, in fact, introduced fragmentation to maternity care rather than reducing it.^17^

Both the political context in which the policy proposal of universal provision of MCOC was implemented in England and the voices of midwives concerned about its impact have been absent from the research recommending the targeted provision of MCOC (Middlemiss et al. 2024). If continuity of carer is to be sustainable, however, researchers and policy makers must consider the concerns raised by many midwives about the negative impact of flexible working practices required to provide intrapartum continuity. Ironically, if MCOC has been promoted as the solution to the problem of women not being listened to, surprise over the difficulties of its implementation is due to a failure to listen to midwives as an undervalued female profession. The pilot studies reported within the literature on the potential for MCOC to reduce inequalities in health care demonstrate how listening to birthing people is fundamental to improving experience and outcomes for those most at risk. They also demonstrate how acknowledging the role of the social determinants of health in working with families can reduce the likelihood of shaming and disrespectful encounters when accessing healthcare. However, as I will argue in the concluding section, by representing the problem in terms of fragmented care, the policy proposal of MCOC presupposes that perinatal health inequalities can be addressed by changing how we work as midwives, without also transforming the context in which we work, which includes national policies that systematically impoverish, stigmatize, and blame individuals for their own suffering.

## Representing the problem of inequalities in perinatal health as health injustice

The time to act on health inequality in England is long overdue and providing rights-respecting maternity care for those most at risk is integral to this. The perinatal period is a critical time for effective intervention to address health inequality, which is known to positively protect mental and physical health for both parent and child (APPG Birth Trauma 2024; Bauer et al. 2014; Horton and Astudillo 2014; Kluny and Dillard 2022). This is unavailable in many parts of the world and, increasingly, only available to some in England (Hoope-Bender et al. 2014).^18^ As we have seen, however, because it represents the problem in terms of fragmented care, the policy proposal of MCOC *presupposes* that perinatal health inequalities can be addressed, to a considerable extent, through reorganization of working practices within the NHS. Consequently, a significant *effect* of MCOC as a policy proposal is to place the burden of addressing health inequalities on midwives by relying on us to work flexibly to deliver intrapartum continuity. In this concluding section, I discuss how the policy drivers that contribute to widening inequalities in perinatal health are often *left unproblematic* when the problem is represented in terms of fragmented care. Moreover, I consider how responses to the problem of perinatal health inequalities might differ if the *problem were represented otherwise* as a matter of health injustice (Bacchi 1999, p. 13).

Representing the policy problem as a matter of fragmented care tends to leave unproblematic the role of policymakers in driving inequalities in health through wider policies, such as austerity and the hostile environment. Inequality in maternal and perinatal deaths is widening in the context of stalling life expectancy, which has declined for women living in the most deprived 10% of areas in England (Marmot et al. 2020). The devastating impact of maternal and perinatal deaths therefore needs to be understood in the context of long-term unequal exposure to adversity and discrimination sustained over the life-course that is making some birthing people sicker and shortening their lives (Delap and Kitchen 2023). Crucially, the UK policy environment continues to shape public and institutional attitudes where individuals are responsibilized for their own disadvantage and the lives of members of marginalized and stigmatized groups are not valued as highly as those of others. Representing the problem in terms of the fragmented healthcare system means that the wider policy conversation that follows from reports of worsening inequality in perinatal outcomes tends to focus on the health care aspects rather than the social determinants that expose a person to high-risk pregnancy. Despite robust and well-known evidence that women and birthing people are at risk of adverse perinatal outcomes because of their socioeconomic circumstances and exposure to structural racism and discrimination (Birthrights 2022; Jardine et al. 2021; Knight et al. 2022), data suggests that health inequality is not recognized as a priority among healthcare professionals and service users and that greater public and professional awareness is needed.

When the problem is represented as fragmented care, the policy of austerity that has been driving inequalities in perinatal health is left unproblematic (Arrieta et al. 2022; Marmot et al. 2020; Martin et al. 2021; Walsh et al. 2022). By relying on midwives to mitigate the effects of social adversity, MCOC does not challenge the wider policy context within which midwives work, which includes the dehumanizing impact of austerity (Clayton et al. 2022, p. 2). While recognizing that ‘healthcare can never fully compensate for the impact of socioeconomic health disparities,’ proponents of MCOC nonetheless insist that NHS midwives are in a ‘unique position to uncover, understand, and act on maternal health inequalities’ (Clayton et al. 2022, p. 2). Yet, prescribing MCOC for the most marginalized, as a means of mitigating social adversity, implies that more ambitious system wide change is not achievable. Instead, the birthing person must rely on the protection and voice of a known midwife to be heard and respected at the time of birth. However, as we have seen by attending to the political and structural context in which it emerged, MCOC’s emphasis on personalized care is not only compatible but consistent with the politics of austerity. Yet austerity is known to have exacerbated health inequalities by reducing social welfare, placing midwives in impossible situations as those essential sources of support that MCOC is expected to enhance access to (including housing, trauma-informed therapies, early years education, health visiting, children’s social care and support for those fleeing domestic violence) are withdrawn (Delap and Kitchen 2023; Walsh et al. 2022).

The hostile environment is another policy driving inequalities in perinatal health, which is left unproblematic when the problem is represented as fragmented care (Delap and Kitchen 2023; Lokugamage et al. 2022; Stevens et al. 2024).^19^ NHS charging for those unable to demonstrate a right to reside in the UK drives inequalities in perinatal health because it disproportionality affects some of the most disadvantaged birthing people living in the UK, exacerbating poorer health outcomes among those who are already socially and economically vulnerable (Birthrights 2022; Pellegrino et al. 2021). Maternity charging has been found not only to deter women from attending for care and to contribute to high levels of stress and anxiety due to (often erroneously attributed) charges and the difficulties in resolving them; it also normalizes racism through the political discourse that surrounds the hostile environment and directly contributes to racial discrimination within the NHS by encouraging healthcare professionals to question whether racially minoritized people are entitled to care (Pellegrino et al. 2021; Rassa et al. 2023). Every day and cumulative experiences of being treated as less than equal contribute to accelerated aging or ‘weathering’ (Gee et al. 2019; Fernandez Turienzo et al. 2021b). In the context of perinatal outcomes, weathering materializes in the body through increased risk of diabetes, hypertension, obesity, and poor mental health, all of which increase the risk of adverse perinatal outcomes (Pellegrino et al. 2021). The policy of MCOC promotes midwives’ role in protecting birthing people against disrespectful care. Yet the deputization of immigration control through maternity charging makes midwives complicit in the weathering of racially minoritized people, whose human right to safe maternity care the policy of the hostile environment deliberately jeopardizes (Birthrights 2022).

How might policy priorities differ if the problem of inequalities in perinatal health was represented as a matter of health injustice rather than fragmented care? Systemic failures within maternity care identified in many reports clearly need to be addressed by enhancing the quality of maternity care within the NHS. However, a health justice approach begins from the recognition that just 10–20% of the disparities in people’s health outcomes are explained by differential access to health care whereas material conditions (including environment, socioeconomic status, and housing) determine health and life expectancy (Thomas 2022; xiii). As Delap and Kitchen (2023, 1) emphasize, where we fail to recognize and accept ‘that structures and practices of our society attach greater value to the rights of some pregnant women, mothers and their children than others, we cannot hope to start to narrow the inequalities in health outcomes.’ We therefore need to resist the tendency within healthcare practice and policy to individualize health behaviours and risks, which work to further shame birthing people and, in turn, to undermine health, discourage engagement and to further silence those who are disadvantaged. Any adequate policy response demands not only that midwifery is properly resourced (so that we can spend sufficient time with birthing people) and that wider support services are available (so that midwives are able to refer birthing people to them) but that *all* health care professionals develop shame sensitive practices that recognize how the promotion of healthy choices can be stigmatizing when it ignores the wider determinants of healthy behaviours (Lokugamage et al. 2022).

The rights-respecting care demanded when inequalities in perinatal health are recognized as health injustice requires care informed by structural competency. This involves consciously working to reduce experiences of shame by recognizing its structural causes, listening without judgment, and resisting personal blame and responsibility. The latter can be done by drawing connections and articulating social causes of ill health and inequality. As Marmot (2015) shows, the impact of inequality on health goes beyond the effect of poverty to include the experience of disempowerment associated with ranking low in a social hierarchy (see also Pickett and Wilkinson 2010). How we talk about the wider determinants of health can actively reduce the experience of shame and encourage service users to engage and disclose concerns. The experience of having little control over one’s life is internalized as personal failure and leads to negative feelings about self, such as shame and guilt, as well as to negative thoughts and expectations about the future. Understanding the ideological function of stigma in reproducing social inequality can help us to rethink how we respond to inequalities in perinatal health (Tyler 2022). When our practice is informed by structural competency, midwives can help to shift the way we think about health and the interventions required to improve outcomes and reduce inequalities by disseminating unambiguous evidence of the wider determinants making people sick and shortening lives.

An adequate policy response equally demands the provision of rights-respecting care based on cultural safety. Lokugamage et al. (2023) discuss, cultural safety is an antiracist, decolonial social justice strategy that is distinct from cultural competency in that it is based on structural reflexivity about power imbalances in therapeutic relationships and healthcare encounters. As such cultural safety requires the recognition of social and colonial origins of health inequalities. This requires professionals to acknowledge their own position of privilege and enable birthing people to determine whether a healthcare encounter or relationship is culturally safe (Lokugamage et al. 2023, 246). Beyond the provision of continuity of care, translating cultural safety into practice in the UK requires active listening to minoritized people’s experiences of healthcare inequalities, the provision of adequately funded interpreter services, addressing racism towards staff and including decolonizing history of healthcare within training (Lokugamage et al. 2023, 347–349).

The mitigation of social adversity cannot be achieved through care and compassion alone. Recognising stigma as an exercise of power indicates that answers to stigma and discrimination associated with health inequalities also need to be found up-stream, beyond individual or community level interventions, which tend to focus on the psychological dimension of stigma. In doing so, we move from a focus on efforts to ameliorate the harmful effects of stigma politics on individuals and communities, towards structural interventions that begin from the social, political, and economic causes of stigma. As Tyler observes (2022, 209), stigma production cascades into our everyday interactions with each other, including our evaluative judgments of requests made by others for care and respect. However, everyday practices can also be turned into sites of struggle, indicating the significant role of health care encounters in tackling inequality and promoting the aim of health justice.

How we represent the problem of inequalities in perinatal health affects the level of public support for policy interventions needed to address the drivers of health inequalities. Representing the problem as health injustice demands that we address the drivers of perinatal health inequalities beyond the healthcare system as a matter of human rights. It focuses attention on how chronic exposure to social adversity and discrimination shapes access, outcomes, and experiences during the critical time of the perinatal period. And it directs attention to the wider determinants of health, which include lack of access to safe maternity care in the context of the NHS staffing crisis, the risks and suffering associated with pregnancy and parenting in poverty, incarceration in pregnancy and following birth, a lack of family support services and overwhelmed mental health services, all of which are driving crisis level interventions by children’s social care and a significant rise in the numbers of children taken into care (Delap and Kitchen 2023).

So long as the focus for addressing inequalities in perinatal access, experience and outcomes remains MCOC (as exemplified in Core20PLUS5 and the RCM Maternity Disadvantage Assessment Tool), in contrast, we risk allowing policy-makers to appear to address the urgent issue of inequalities in perinatal care while calls to tackle structural racism and social inequality within and beyond the healthcare system remain unaddressed. It might be objected that the implementation of MCOC was never intended as a singular solution to the complex challenge of perinatal health inequalities but only one aspect of a wider policy drive to reduce health and social inequalities. By calling attention to policy-making as a productive activity, however, I have sought to make visible the politics of academic knowledge and research. As such, the policy analysis presented here is not an argument against relational ways of working but draws critical attention to the material effects of all policy proposals, which necessarily represent problems in particular ways while obscuring others.

It has become standard practice to reference the social determinants of health in relation to addressing health inequalities. For instance, the foreword to the Equity and Equality Guidance for Local Maternity Systems states that ‘if we are to achieve equity, even more needs to be done to address the social determinants of health’ and refers to the NHS Long Term Plan’s recognition that ‘the public, private and third sector need a greater focus on the social determinants of health’ (NHS England 2021c, 4 citing NHS England 2019, 33) Nevertheless, interventions pursued by government, including the Long Term Plan, persist in focusing on individual behaviours and models of care. This phenomenon is referred to by public policy researchers as life-style drift (Halsall et al. 2024). Given this tendency, if policy action on the drivers of inequality is to become a priority, we need to raise awareness among the public and all those working in perinatal care about the impact of inequality on perinatal outcomes. The Health Foundation has called for a different and more powerful story, which reframes how we talk about health, away from individual behaviour and disease toward recognizing its wider structural determinants (L’hôte et al. 2022). As health professionals, we are responsible for calling out the causes of avoidable ill health and premature deaths. This includes using the trust and authority we are granted as health care professionals to communicate the structural causes of the suffering we encounter to the people we care for.

## Data Availability

All primary sources drawn on for this review paper are available online via URLs included in the list of references.

## Footnotes

1 Developed in response to the finding that social risk factors were not consistently assessed and reported (Felker et al. 2024), the Maternity Disadvantage Assessment Tool (MatDAT) identifies four distinct levels of care based on social and care needs. MatDAT recommends antenatal and postnatal continuity from a named midwife for birthing people who are thriving and whose needs are met by universal services (level 1). Beyond thriving families, at levels 2, 3 & 4, the midwifery response to social risk factors is MCOC, which requires intrapartum continuity.

2 While MCOC can be delivered by a team of midwives, most birthing people have 7–9 appointments antenatally. In practice, this means there is often a trade-off between ensuring intrapartum continuity and providing ante-natal continuity.

3 The findings of Rayment-Jones et al. (2023), in which they refine this hypothesis are discussed at length below. The implications of representing the problem of health inequality in terms of fragmented care is apparent in how the model of *integrated* care based on MCOC is represented in Fig. 2. In this image, based on three concentric circles, the MCOC team in the centre ‘act as a single point of contact for multi-disciplinary services described in the middle layer. The location can help to foster a sense of belonging to the community and encourage use of other services and facilities. The middle circle [which includes food banks, charities and social workers, among other things] represents *integrated* care with other forms of local support that can be easily accessed on a needs-led basis. The outer circle [which includes the Home Office and Housing, among other things] represents specialist services that can be accessed through clear referral pathways’ (Rayment-Jones et al. 2023; e324).

4 Findings from the other six UK-based trials were published in 1989, 1993, 1996, 2000, 2003 and 2003. The other 10 trials reviewed were conducted in Australia (6), Ireland (1), China (1), Canada (1) and Sweden (1).

5 Missed opportunities consistently feature in reports on stillbirths and enquiries into why mothers and babies die (Draper et al. 2015; Kirkup 2015, 2022; Independent Maternity Review 2022). This includes reports of dehumanised care and examples of women’s basic rights to dignity and consent in birth not being met (Scott and Bevan 2009; Birthrights 2013, 2022). Women report concerns about being left alone while in labour and about failures to share important information with them (Scott and Bevan 2009).

6 For instance, among the examples of existing continuity of care services listed in the report were the One-to-one Limited-multicentre in Liverpool where ‘each midwife will be on call for women in their caseload most of the time but they are encouraged to protect at least one day a week by diverting their phone calls to their partner midwife’ and St Mary’s caseload team for women with social risk factors where midwives ‘will be on call overnight three to four times a week to increase continuity of intrapartum care and have two full days off’ (Sandall et al. 2016a, p. 34, 32).

7 Julia Cumberlege is a Conservative Party politician who served as Junior Health Minister in 1992 and now sits as a Conservative Life Peer in the House of Lords. As Ashley (2016) points out, Cumberlege was well-known for chairing *Changing Childbirth* in 1993, which established a new vision for the transformation of maternity services in terms of ‘continuity of care, choice and control’ (Department of Health 1993; Middlemiss et al. 2024, p. 9). Addressing the House of Lords in 2000, Cumberlege said ‘we are all trapped in a marvellously pure ideology, the ideal socialist dream’ and she went on to argue against the requirement that the NHS be free at the point of use (Hansard 2000). Moreover, Cumberlege was the founder of Cumberlege Connections (now Eden and Partners), a specialist consultancy company to the health sector, which partnered with Price Waterhouse Coopers, which successfully bid for contracts with Clinical Commissioning Groups.

8 In launching the policy, then Health and Social Care secretary, Jeremy Hunt declared, ‘There are few moments in life that matter more than the birth of a child, so the next step in my mission to transform safety standards is a drive to give mums dedicated midwives who can get to know them personally and oversee their whole journey from pregnancy to labour to new parent’ (Press Association, 2018). In doing so, Hunt contributed to a policy narrative which presents the midwife’s personal and dedicated relationship to women as the way to address safety standards and increase responsiveness of maternity services.

9 Crowther (2017, 3) moves from acknowledging the concerns of midwives, many of which relate to the extensive demand for flexibility, to suggesting that we as midwives have a moral responsibility to implement MCOC. ‘Joy and love of practice’ are contrasted with resistance to change. In her dismissal of midwives’ concerns regarding MCOC, Crowther provides anecdotal evidence of her own love of working within various models of practice, including as a self-employed independent midwife and as a caseload rural midwife in New Zealand, experiences which she describes as lifechanging. Employing gendered language, Crowther creates the image of the midwife realising herself through her work. Her choice of words, including flexibility, willingness, positivity, generosity, love and joy rely on and reinforce the feminine forms of emotional labour expected of women as caregivers.

10 The head of the midwives’ UK trade union and professional organisation, for instance, writes in the foreword to *Implementing Better Births: Continuity of Carer*, that the model proposed provides ‘well documented benefits…. to midwives’ role satisfaction and personal development’ (Gill Walton in NHS England 2017).

11 In support of MCOC, they quote one midwife feeling ‘up against the machine of the NHS’ and contrast this to the sense of personal responsibility enjoyed by midwives working within MCOC (Finlay and Sandall 2009, 1229-30).

12 *Better Births* recognized that midwives are more likely to feel pressured at work than other NHS staff, with almost half reporting having suffered from work related stress (NHS England 2017, 27). Moreover, an NHS survey found that only a third of all midwives would be willing to work in a continuity-based model that included intrapartum care across all settings (Taylor et al. 2019, p. 4). Nevertheless, *Better Births* acknowledged the ‘inconvenience to midwives of being on stand-by and called out at unsociable hours’ while recognizing that ‘translating the ideas set out into reality will have to be done by commissioners, managers, and health professionals with an understanding of local opportunities and challenges’ (NHS England 2017, 5, 84).

13 While they highlight significant concerns relating to maternity workforce stress, sickness, and retention, McInnes et al. (2020, 2) highlight trusting relationships as a key mechanism for driving up midwife commitment to MCOC. This is identified as fundamental to overcoming barriers to continuity working, which, they highlight, have so far only been small scale, slow to grow or not sustained (McInnes et al. 2020, p. 2).

14 Using an anonymous online survey, Taylor et al. (2019) found that many midwives were unable or unwilling to work in continuity models and that this was most often related to the requirements for being on call and available for intrapartum care. Qualitative analysis highlighted several barriers including and most commonly childcare responsibilities, but also wellbeing and work-life balance. In this study, midwives predominantly described MCOC as having a negative impact on working lives.

15 A website that promoted the Jubilee team’s service provided an account of ‘a day in the life’ of a community midwife, which captures the enjoyment in self-sacrifice that advocates of MCOC promote. Here, a working day starts with a text message at 08.30 and ends with a ‘yay’ and a smiley face after returning home at 04.15. In between these hours there is a visit to a friend, which ‘ends up being a “put the world to rights” visit’ taking up most of the afternoon, a return home to watch TV at 18.00 and time in bed between 22.00 and being called out at 23.55. Luckily, as she writes, ‘the sky is clear and the stars are bright’ and she appears cheerful throughout, as she recounts arriving home after 4am ‘with a smile due to the feeling of helping a lady to birth her baby at home.’

16 The meeting was attended by the Head of Maternity, Children and Young People at NHS England and National Maternity Safety Champion for the Department of Health and by the Deputy Head of Maternity Services for NHS England.

17 For instance, because midwives were not employed by the hospital trust, when women who had booked under Neighbourhood Midwives required hospital care, their continuity midwife would accompany the woman but relied on hospital midwives to take over responsibility for care. This fragmentation in service provision was associated with complex financial arrangements, which led to the service being expensive and unsustainable, with many birthing people left without a midwife late in pregnancy, albeit having been offered continuity if they could afford to pay privately.

18 A Care Quality Commission (2023) survey of more than 21,000 birthing people in 2022 found less than two thirds of respondents reported being able to always access a member of staff to help them while in labour and during birth. Close to half reported not being able to access help when needed during their postnatal admission.

19 Formally announced in 2012, the hostile environment refers to all policies designed to make life difficult for people unable to demonstrate their right to reside in the UK. NHS charging (and maternity charging, in particular) is one of several ways in which border control was deputized in the 2014 Immigration Act by making midwives (among other health care professionals and ordinary citizens) responsible for surveillance, detection and enforcement of immigration controls, including by restricting access to maternity care (Griffiths and Yeo 2021).
